# Anion States and Dissociation Pathways of the Universal Radiosensitizer Model 2,4-Dichloro-5-nitropyrimidine

**DOI:** 10.1021/acs.jpca.6c00754

**Published:** 2026-08-10

**Authors:** Ely G. F. de Miranda, Marcio T. do N. Varella

**Affiliations:** Instituto de Física, Universidade de São Paulo, Rua do Matão 1371, 05508-090 São Paulo, São Paulo, Brazil

## Abstract

We present a computational investigation into the anion states of 2,4-dichloro-5-nitropyrimidine (NDP), focusing on dissociative electron attachment pathways motivated by recent mass spectrometry experiments. The molecule exhibits a unique combination of low-energy electron-induced mechanisms potentially relevant to synergistic effects in concurrent chemo-radiation cancer therapy. Our calculations reveal two bound states (π_1_^*^ and π_2_^*^), three shape resonances (π_3_^*^, σ_CCl1_^*^, and σ_CCl2_^*^), and a π_4_^*^ resonance of mixed shape and core-excited character. We attribute the intense fragmentation, [M–NO]^−^ and [M–NO_2_]^−^, and the parent anion state at 0 eV mainly to the π_3_^*^ state, characterized as either a weakly bound state or a resonance slightly above threshold. While such processes could in principle occur via vibrational Feshbach resonances, the π_2_^*^ state is too deeply bound (at least by 0.71 eV) to enable this mechanism. The complex fragmentation induced by nearly zero energy electrons would instead arise from nonadiabatic interactions between the π_3_^*^ state and the lower-lying bound states π_1_^*^ and π_2_^*^. This coupling between bound anion states and a low-energy resonance creates pathways for internal conversion and dissociation and could be viewed as a target property for radiosensitization.

## Graphical abstract

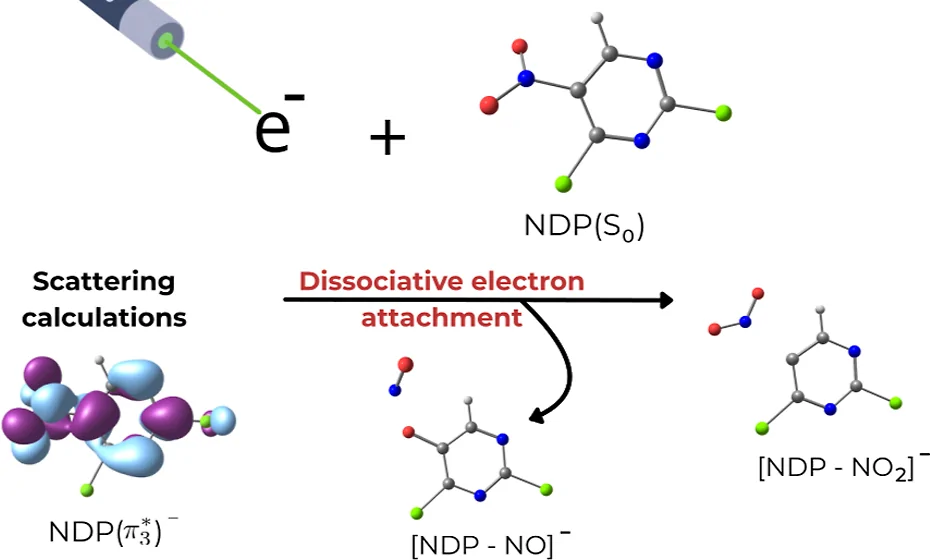

## Introduction

Over the past two decades, the importance of electron–molecule interactions, especially the formation of transient anions through electronic capture, has become increasingly evident.^1^ These interactions play pivotal roles in various biological processes, including radioactive damage to DNA and biomolecules,^2–4^ cancer treatments involving hadron and radio-chemical therapies,^5–7^ electron transfer chains from cell respiration,^8–10^ olfactory recognition,^11^ drug activity including antioxidants,^10,12–16^ and toxicity mechanisms.^1,17^

Strong electron acceptors can capture electrons under reductive conditions in cells, generating transient negative ions (TNIs) or resonance. This process initiates vibrational dynamics that lead to chemical bond breakage, producing reactive species, which are often radicals. This process is termed dissociative electron attachment (DEA). The formation of resonances may thus induce lesions and other deleterious effects on biomolecules in their cellular environment.

In clinical radiotherapy, a major challenge is overcoming the radioresistance of cancer cells, particularly those in hypoxic regions of tumors, which can be 2–3 times more resistant to ionizing radiation than normoxic cells.^18^ To address this, chemical radiosensitizers are employed to enhance tumor cell inactivation.^6^ A key mechanism involves electron-affinic sensitizers, which efficiently capture low-energy secondary electrons generated by radiation.^19^ Upon capture, these compounds can undergo DEA, leading to the formation of highly reactive radicals that cause lethal DNA lesions, such as strand breaks and base fragmentation. This DEA-mediated pathway is a crucial mechanism for several potent radiosensitizers whose high electron affinity promotes the formation of deleterious TNIs.^18^ Beyond direct DEA, similar electron-induced reactions can also play a role in the chemo-radiation treatment of cancer, possibly due to inhibition of DNA repair, among other mechanisms,^20^ such as associative electron attachment (AEA)^21^ and DNA sensitization.

Several molecules, including some with high electron affinity functional groups, have been utilized or proposed as radiosensitizers in cancer therapy. This has motivated efforts to characterize the TNIs of compounds with radiosensitizing potential.^10,15,22–27^ Much of this theoretical effort has historically focused on halogenated biomolecular analogues, where the coupling between ring-localized π* anion states and dissociative σ*­(C–X) orbitals provides a well-established pathway for the DEA-driven release of reactive radicals that underpins radiosensitization.^15,23–25^ Compounds combining several distinct electron-accepting motifs within a single molecular scaffold, however, can support a richer manifold of anion-mediated reactions and therefore represent a new paradigm for the rational design of radiosensitizers. In this context, 2,4-dichloro-5-nitropyrimidine (NDP) was recently highlighted as a universal molecular model for understanding electron-induced elementary processes underlying radiosensitizing activity,^28^ since the molecule exhibits a striking variety of dissociation fragments associated with low-energy electrons, combining in a single compound practically all known modes of action of low-energy electrons hypothesized to cause synergy in concurrent chemo-radiation therapy: DEA, AEA (with an exceptionally long-lived parent anion of ∼1.2 ms^28^), high electron affinity enhancing energy transfer, and potential DNA sensitization through its pyrimidine scaffold. Despite its toxicity, which raises questions about its practical use as a drug, the bioactivity of NDP could be due to several modes of action. The molecule presents a unique combination of strong electron-accepting motifs, namely, the nitro group, chlorine atoms, and the unsaturated pyrimidine ring (see Figure 1). The presence of these multiple electron-accepting sites produces a rich spectrum of anionic states and an interplay between DEA and AEA, among other possible reaction channels.^28^ The combination of bound and TNI states, along with the complex reaction dynamics triggered by those states, poses a challenge to computational models.

**Figure 1:**
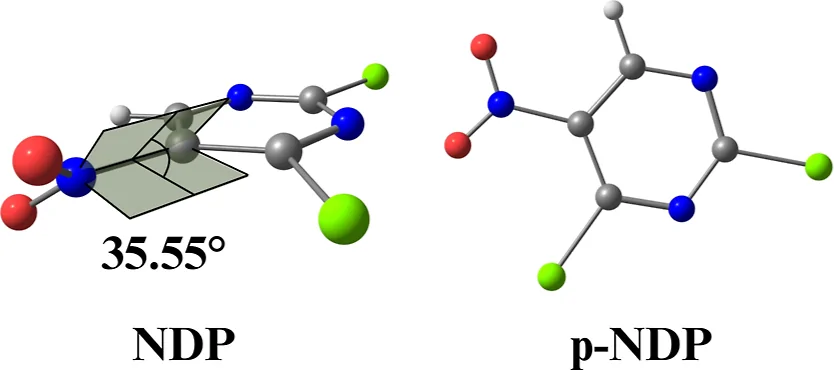
NDP and *p*-NDP geometries. The oxygen atoms are indicated in red, carbon in gray, nitrogen in blue, chlorine in green, and hydrogen in white.

Despite experimental progress in characterizing NDP’s radiosensitizing activity,^29^ theoretical studies remain scarce. In particular, no theoretical characterization of the anion states of NDP has been reported to date. The experimental study by Luxford et al.^28^ provided DEA spectra, parent anion lifetime, and electron affinity measurements, but the electronic states underlying the observed fragmentation, in particular the intense zero-energy signal, have not been identified or rationalized.

In this work, we employ a combination of scattering and bound-state techniques along with thermodynamic analysis to provide the first theoretical description of the anion states of the NDP molecule and to gain insights into the stabilization and dissociation mechanisms. More broadly, a better understanding of the reaction pathways arising from anion states associated with specific functional groups could aid in proposing other universal radiosensitizers, and the coupling between bound anion states and a low-energy resonance may constitute a target molecular property for the design of radiosensitization.

This paper is structured as follows: The Computational Procedures section outlines the theoretical and computational methods for scattering calculations, specifically the Schwinger multichannel method with pseudopotentials (SMCPP) for TNI characterization. The Results and Discussion sections present our calculations and discuss the findings, respectively, while the Conclusions section summarizes the conclusions.

## Computational Procedures

Equilibrium geometries and vibrational analysis for the neutral molecules were performed with density functional theory (DFT), employing the ωB97XD functional and the aug-cc-pVDZ basis set, as implemented in the Gaussian09 package.^30^

Although the NDP molecule is not planar (torsion angle indicated in Figure 1), we also optimized the ground state, enforcing a planar geometry, referred to as *p*-NDP, also shown in Figure 1. This procedure, which was used in previous studies,^25,27,31^ allows the symmetry decomposition of the cross sections, making the signature of resonance states much clearer, due to the reduced background, and saving considerable computation time. We are not interested in the scattering cross sections per se; they mainly serve as techniques to obtain both the energy and autoionization lifetimes of the TNIs. The error introduced by geometry planarization can be much larger for particular anion states of NDP compared with other small biomolecules (see below).

The scattering calculations were performed in the fixed-nuclei approximation. The target ground state and the scattering calculations were carried out by replacing the nuclei and core electrons of the heavier atoms (all but hydrogen) by the norm-conserving pseudopotentials of Bachelet, Hamann, and Schlüter (BHS).^32^ The electronic ground state was described at the restricted Hartree–Fock level employing Cartesian Gaussian basis sets generated according to Bettega et al.,^33^ which are suitable for use with the BHS pseudopotentials. The 6s5p2d basis set was employed for chlorine, and the 5s5p2d basis set for nitrogen, carbon, and oxygen atoms,^33^ while the 4s basis set reported by Dunning^34^ was chosen for hydrogens, amounting to a total of 357 basis functions for each structure. The RHF calculations were performed with the GAMESS package.^35^ The basis set exponents are listed in the “Scattering details” section of the Supporting Information.

The scattering cross sections were computed with the parallel version of the Schwinger Multichannel method (SMC) implemented with the Bachelet–Hamann–Schlüter pseudopotentials (SMCPP).^36,37^ The technique was recently reviewed,^38^ so we briefly describe some relevant aspects. The scattering electronic wave function is given by

1 $$|\Psi_{k_{i}}^{\left(\pm \right)}\rangle =\sum_{\mu }c_{\mu }^{\left(\pm \right)}\left(k_{i}\right)|\chi_{\mu }\rangle$$

where **k**_*i*_ denotes the wave vector of the incident electron and + (−) stands for the boundary condition associated with outgoing (incoming) spherical waves.^39^ The method uses square-integrable functions to build the trial set {χ_μ_}, which is very computationally convenient. The basis vectors |χ_μ_⟩ are referred to as configuration state functions (CSFs) and correspond to spin-adapted (*N* + 1)-particle Slater determinants built on the closed-shell ground state of the neutral target, comprising *N* electrons.

The scattering calculations were restricted to the elastic channel, a reasonable approximation for electron energies below the excitation threshold. Two models were explored to construct the CSF space. In the static-exchange (SE) approximation, the target is kept frozen in the ground state |Φ_0_⟩. At the same time, the unoccupied (virtual) orbitals are used as scattering orbitals |ϕ_*m*_⟩, i.e., $|\chi_{m}\rangle =A_{N+1}|\Phi_{0}\rangle ⊗|\phi_{m}\rangle$ , where $A_{N+1}$ is the antisymmetrization operator. This approximation does not recover the response of the target electrons to the incoming electron. Even though the SE approach retrieves the physics of shape resonances, at lower energies, the dynamical response of the target electrons, referred to as correlation-polarization effects, should be accounted for. In the static-exchange plus polarization (SEP) approximation, virtual excitations of the target are allowed, so the CSF space is augmented with configurations of the type $|\chi_{im}\rangle =A_{N+1}|\Phi_{i}\rangle ⊗|\phi_{m}\rangle$ , in which |Φ_*i*_⟩ represents a single excitation of the target and |ϕ_*m*_⟩ is a scattering orbital.

The SEP functions require the choice of three orbitals, two of which are associated with the excitation of the target (hole and particle), and the third is the scattering orbital. The CSF space is built according to the energy criterion proposed by Kossoski and Bettega.^40^ All single-particle orbitals whose energy eigenvalues satisfy ϵ_scat_ + ϵ_part_ – ϵ_hole_ < Δ are taken into account, where ϵ_scat_, ϵ_part_, and ϵ_hole_ are the energies of the scattering, particle, and hole orbitals, respectively, while Δ is a cutoff. Finally, modified virtual orbitals (MVOs)^41^ were used as particle and scattering orbitals. They were generated from cationic Fock operators with charges of +6 for NDP and +2 for *p*-NDP.

The integral cross sections (ICSs) of *p*-NDP were decomposed into the *A*′ and *A*″ components of the C_S_ group, while for the NDP, the ICSs are not decomposed since they lack a symmetry to explore. In the SEP approximation for *p*-NDP, we employed Δ = 0.45 hartree for the *A*′ and *A*″ components, generating 16,501 and 16,516 configurations, respectively. Similarly, we employed Δ = −1.20 hartree for NDP, resulting in 16,172 configurations. Providing a balanced description of polarization effects in SEP for systems with several resonances is challenging. In the absence of electron transmission spectroscopy (ETS) data for NDP, the multimethod bound anion state energies reported in Table 1 (roughly −1.3 to −1.7 eV for π_1_^*^) were used semiquantitatively to guide the choice of the SEP cutoff Δ, so that the lowest π* state emerges bound and within a physically reasonable range of these values; we rely on these calculations for the existence, ordering, and relative shifts of the bound π* states rather than their absolute energies.

**Table 1:** Vertical Energies (in eV) of the Bound Anion States π_1_^*^ and π_2_^*^ for the NDP and *p*-NDP Structures^a^

state	method	NDP	*p*-NDP
π_1_^*^	CAM-B3LYP	–1.66	–1.11
	ωB97XD	–1.57	–1.05
	CASPT2	–1.29	–0.89
	SMCPP	–1.82	–1.25
π_2_^*^	CAM-B3LYP	–0.95	–0.83
	ωB97XD	–0.92	–0.78
	CASPT2	–0.71	–0.66
	SMCPP	–1.50	–1.08

a The estimates were obtained with TDDFT using the functionals indicated in the table, CASPT2, and SMCPP (SEP approximation). See text for details.

No corrections were used to account for the long-range dipole potential contribution to the higher partial waves. This contribution would impact only the background cross section and obscure the resonance states of interest. The primary objective is to acquire the TNI spectra; therefore, symmetry-resolved cross sections are reported without correcting higher partial waves. It should be clear, however, that cross-sectional magnitudes are underestimated.

The scattering Hamiltonian represented in the square-integrable CSF basis of the SMCPP calculation can be diagonalized. This procedure generates a set of pseudo-states, since the actual spectrum is not discrete, but it provides insight into the resonance pseudo-orbitals. Therefore, the pseudo-orbitals characterizing the formation of anion states can be inferred from the pseudo-states, both in the SE and SEP approximations. To construct those pseudo-orbitals, we use the pseudo-states obtained from the SE (static-exchange) and SEP approximations. The pseudo-orbital is built from the linear combination of CSFs that define the resonance-like pseudo-state. For the SEP approximation, we project the CSFs onto the SE subspace.

The lower excited states of the anion were also explored with the MS-CASPT2 method.^42^ These calculations were performed with OpenMOLCAS software;^43^ the active space and further technical details of the CASSCF/CASPT2 calculations are provided in the Supporting Information. The bound-state DFT, TDDFT, and CASPT2 calculations were performed using the cc-pVDZ basis set for C and H atoms, while the aug-cc-pVDZ basis set was used for N, O, and Cl atoms. This choice was motivated by two aspects: (i) reducing the computational effort and SCF convergence problems, which may arise from near linear dependencies and numerical instability,^44^ and (ii) ensuring a physically meaningful description of the frontier orbitals, as the use of excessively diffuse functions could artificially distort the electron density. This spurious delocalization causes the frontier molecular orbitals to become overly diffuse, which complicates the identification of the relevant π* states and can qualitatively compromise the interpretation of the excited-state spectrum.^45,46^ The dissociation thresholds for the most prominent dissociation products were calculated employing G4­(MP2) methodology.^47^

The energies of the bound states and shape resonances were also estimated from empirically rescaled virtual orbital energies (sVOE) of the neutral molecule, following the protocols of refs 48 and 49 . Two procedures are employed: in *sVOE/HF*, the geometries are optimized at the MP2/6–31G­(d) level and the VOEs are obtained from HF/6–31G­(d) calculations, whereas in *sVOE/DFT*, both the geometry optimization and the VOE estimation are carried out at the B3LYP/6–31G­(d) level. The empirical rescaling was calibrated for π* and σ* resonances.^50–52^ A systematic description of all scaling relations used in this work, together with the corresponding MP2/6–31G­(d) and B3LYP/6–31G­(d) optimized geometries, is provided in the Supporting Information. We stress that the sVOE methodology is a semiempirical approximation with inherent limitations: the scaling relations assume a linear correlation calibrated on a specific training set. The sVOE estimates are therefore presented in this work as an independent cross-check against the more rigorous SMCPP/SEP and multimethod bound-state calculations, not as a primary result.

## Results

### Bound-State Calculations

Since we lack experimental information about the energy of the anion states, we begin our analysis with bound-state calculations. The energies of the vertically bound anion states, which are more stable than the neutral ground state at the optimal geometry of the latter species, are vital for validating our calculations and interpreting the experimental DEA data for NDP. For this reason, we employed different methods to estimate the binding energies of the stable anion states, namely, DFT with several functionals and CASPT2 employing a CASSCF­(14e,14o) wave function (see Supporting Information for details).

For NDP, we found two anion-bound states denoted as π_1_^*^ and π_2_^*^ states since they are formed by electron attachment to those virtual orbitals, as shown in Table 1. According to the DFT calculations, the anion ground state (π_1_^*^) is more stable than the neutral ground state by 1.6–1.7 eV, while SMCPP predicts a slightly more stable anion lying 1.8 eV below the neutral ground state. The convergence of the CASPT2 energy for the anion states was difficult as it required a considerably large active space and a diffuse basis set. The π_1_^*^ state is predicted to be less stable by 0.3–0.5 eV compared to the other methods. For the second bound state (π_2_^*^) of NDP, we performed time-dependent DFT (TDDFT) calculations, in addition to CASPT2 and SMCPP. Note that TDDFT is used here solely for two well-defined purposes: (i) to estimate the energy of the π_2_^*^ bound state (a genuine bound state of the anion, not a resonance) and (ii) to estimate the first electronic excitation threshold of the neutral molecule, which delimits the validity range of the elastic scattering approximation. The discrepancies are somewhat larger, with energies ranging from −1.50 eV to −0.71 eV, although all methods predict strongly bound states. Table 1 also shows results for the *p*-NDP structure. Even though the anion states are consistently less stable for the planarized structure, the same trends are observed for NDP and *p*-NDP.

In addition to the vertical-attachment calculations reported in Table 1, we estimated the adiabatic electron affinity of NDP by optimizing the geometries of both the neutral molecule and its valence anion at the ωB97XD/aug-cc-pVDZ level. The adiabatic electron affinity is defined as EA_adia_ = *E*_neutral_(*R*_neutral_) – *E*_anion_(*R*_anion_) and is reported with the conventional positive sign for a stable anion (EA > 0 ⇔ anion more stable than the neutral); this should be contrasted with the negative vertical energies listed in Tables 1 and 2, which follow the convention *E*_anion_(*R*_neutral_) – *E*_neutral_(*R*_neutral_). The resulting value is +1.94 eV, which lies below the figure of 2.92 eV quoted by Luxford et al.^28^ We emphasize that the latter is not a direct spectroscopic measurement but an inference from the observed anion autodetachment lifetime through a statistical kinetic model; the same authors themselves noted that 2.92 eV is anomalously high compared to analogous nitro-aromatic compounds, and their own B3LYP/6–31 + G­(d) calculation yielded 2.11 eV. Our ωB97XD value is in good agreement with Luxford’s own DFT estimate, and both theoretical estimates suggest that the adiabatic EA lies closer to 1.9–2.1 eV than to 2.9 eV. Beyond that, the optimization reveals a qualitative structural feature of clear mechanistic relevance: the anion is essentially planar, the NO_2_ group rotating from its twisted neutral configuration to align with the molecular plane.

**Table 2:** Resonance Positions, in eV Units, Obtained with Different Methods for the NDP and *p*-NDP Structures^a^

system	method	π_1_^*^	π_2_^*^	π_3_^*^	π_4_^*^	σ_CCl1_^*^	σ_CCl2_^*^
NDP	SMCPP/SE	0.21(≲0.05)	0.87(0.08)	2.39(0.30)	7.12(1.26)	4.93(1.18)	4.93(1.18)
*p*-NDP	SMCPP/SE	0.78(0.08)	0.95(0.18)	4.37(0.92)	7.85(0.97)	4.92(1.93)	4.92(1.93)
NDP	SMCPP/SEP	–1.82	–1.50	0.38(0.05)	3.40(0.34)	1.42(0.55)	2.46 (0.80)
	sVOE/HF	–1.04	–0.62	0.25	3.41	1.29	2.18
	sVOE/DFT	–1.75	–1.12	–0.29	2.65	1.38	1.99
	CASPT2	–1.29	–0.71	0.32			
*p*-NDP	SMCPP/SEP	–1.25	–1.08	2.15(0.71)	4.55 (0.66)	1.78(0.90)	2.94(0.90)
	sVOE/HF	–1.24	–0.59	0.60	3.64	1.32	2.14
	sVOE/DFT	–1.82	–1.11	–0.15	2.76	1.40	1.98
	CASPT2	–0.89	–0.66	1.50			

a For the scattering (SMCPP) calculations, the widths are given in parentheses, also in units of eV. The SE-approximation results are presented for completeness, but are not directly comparable to the other calculations. The SE π_1_^*^ width is narrower than the 0.05 eV grid resolution and is reported as an upper bound. The sVOE/HF and sVOE/DFT π* values were obtained using the scaling relations from refs 48 and 49 , respectively, while the σ_CCl_^*^ values were obtained using the scaling relations specifically calibrated for C–Cl antibonding orbitals in refs 50, 52, and 51 , respectively.

### Scattering Calculations

The calculated cross sections for the *p*-NDP geometry (*C*_s_ group, upper panel) obtained in the SE and SEP approximations are shown in Figure 2. The SE calculations show four peaks and a broad structure. In the *A*″ symmetry component, the peaks are found at 0.78 eV (π_1_^*^), 0.95 eV (π_2_^*^), 4.37 eV (π_3_^*^), and 7.85 eV (π_4_^*^). The broad structure around 4.92 eV in the *A*′ component can be associated with σ_CCl1_^*^ and σ_CCl2_^*^ anion states, where σ_CCl1_^*^ and σ_CCl2_^*^ denote the antibonding orbitals of the C–Cl bond closer to the NO_2_ group and of the one farther from it, respectively. The σ_CCl_^*^ resonances are known to have shorter lifetimes,^23,27,50,53^ thus giving rise to broader peaks in the cross section (see also Table 2).

**Figure 2:**
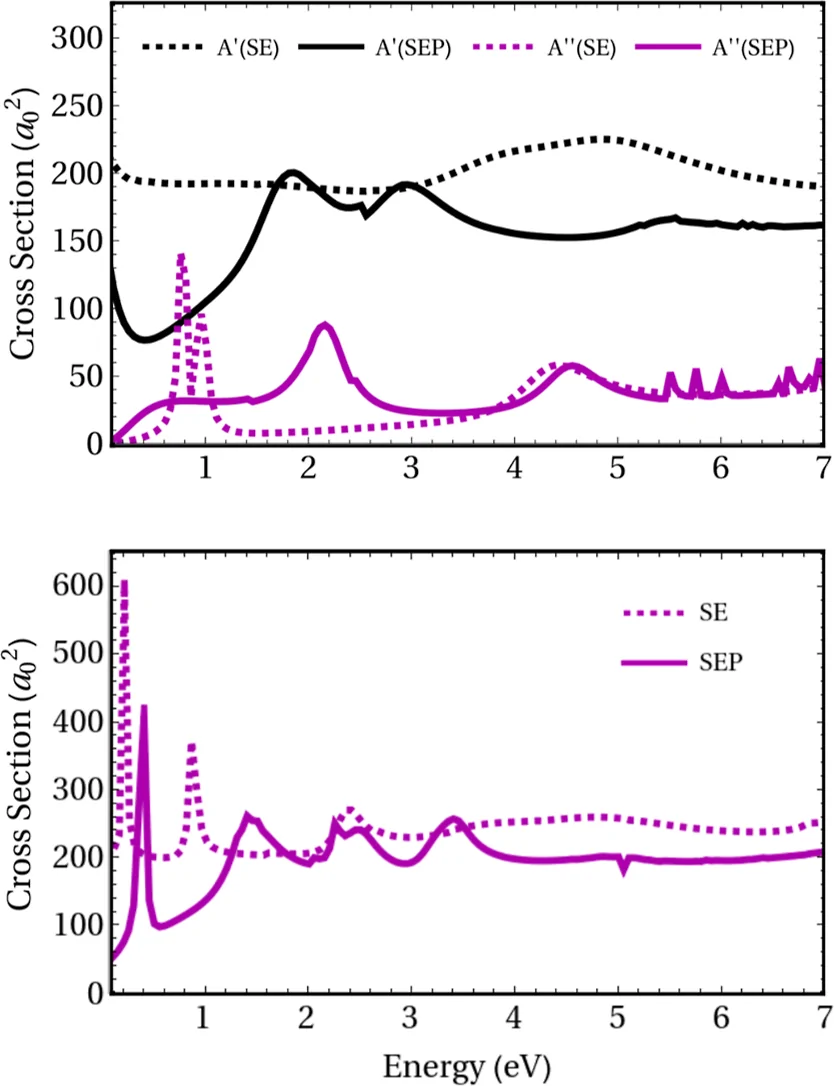
ICS calculated in the SE (dashed lines) and SEP (solid lines) approximations for *p*-NDP (up) and NDP (down). For *p*-NDP, the cross sections are decomposed into the *A*′ and *A*″ symmetry components.

The inclusion of correlation-polarization effects in the SEP approximation shifts the resonances to lower energies, as expected. The π_1_^*^ and π_2_^*^ become bound states with energies of −1.25 eV and −1.08 eV, respectively. The other anion states are located at 2.15 eV (π_3_^*^), 1.78 eV (σ_CCl1_^*^), 2.94 eV (σ_CCl2_^*^), and 4.55 eV (π_4_^*^). The excitation threshold for the first excited triplet state of the neutral molecule, which was estimated at 3.84 eV for *p*-NDP (ωB97XD with the cc-pVDZ basis set for C and H and aug-cc-pVDZ for N, O, and Cl), indicates that π_4_^*^ could acquire mixed shape and core-excited character. Since the cross section was calculated within the elastic (single-channel) approximation, each peak formally corresponds to a shape-type feature; however, because the π_4_^*^ position lies above the first electronic excitation threshold, its observed character is expected to reflect a mixture of shape and core-excited contributions in the actual experiment.

For NDP, the SE approximation gives four peaks and a broad structure located at 0.21 eV (π_1_^*^), 0.87 eV (π_2_^*^), 2.39 eV (π_3_^*^), 7.12 eV (π_4_^*^), and 4.92 eV. The latter broad structure arises from overlapping σ_CCl1_^*^ and σ_CCl2_^*^ states. There is some correspondence between the energies of the anion states for both geometries, except for the π_3_^*^ resonance. We believe this difference stems from the electronic density around the NO_2_ site, as discussed below. The SEP approximation indicates four peaks at 0.38 eV (π_3_^*^), 1.42 eV (σ_CCl1_^*^), 2.46 eV (σ_CCl2_^*^), and 3.40 eV (π_4_^*^). Once more, π_1_^*^ and π_2_^*^ become bound states. The first excited state was estimated at 2.73 eV for NDP (ωB97XD with the cc-pVDZ basis set for C and H and aug-cc-pVDZ for N, O, and Cl), ≈1 eV below the excitation energy obtained for the *p*-NDP structure. Nevertheless, the mixed character of the π_4_^*^ state is also suggested for the NDP structure. Finally, the higher-lying structures above ∼5 eV are spurious resonances that arise from treating energetically accessible (open) electronic states as energetically forbidden (closed) ones in the elastic (single-channel) approximation, i.e., electronic states whose excitation energies lie below the collision energy are not included as scattering channels.

Figure 3 shows the total ICSs of NDP and *p*-NDP in both SE and SEP approximations. The calculated positions and widths are summarized in Table 2. The widths were obtained from local-approximation Breit–Wigner profiles. The resonance widths are compatible with both NDP and *p*-NDP states. The energies of the bound states and shape resonances were also estimated with the two scaled virtual-orbital energy (sVOE) procedures (sVOE/HF and sVOE/DFT) detailed in the Computational Procedures section; the corresponding values are listed in Table 2. For completeness, CASPT2 results are also presented (see below). Since CASPT2 is a bound-state method, it does not describe the resonance as such; the reported energy corresponds to a state stabilized by the finite basis set and carries no associated width. We include it only for the π_3_^*^ state, whose narrow, nearly bound character (Γ ≈ 0.05 eV) makes this a meaningful qualitative cross-check of the SMCPP/SEP results, rather than a rigorous resonance determination.

**Figure 3:**
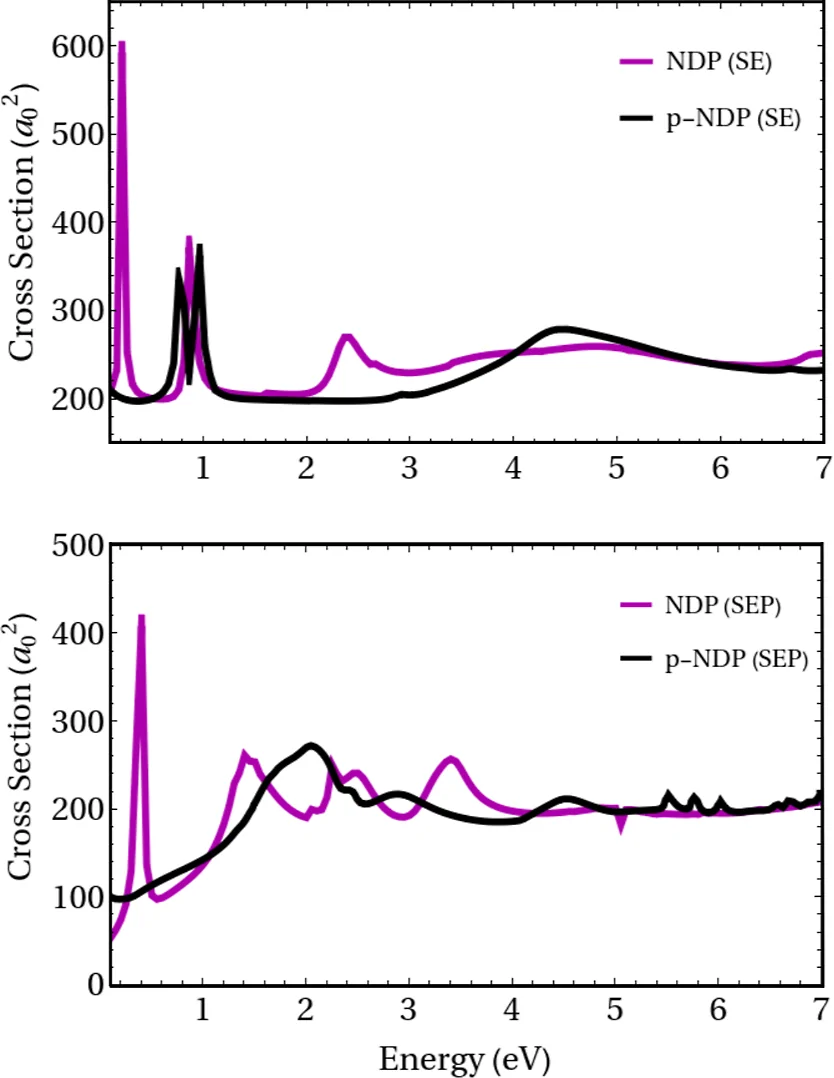
ICS calculated in the SE (up) and SEP (down) approximations for *p*-NDP (black) and NDP (purple).

The positions of the π_3_^*^ and π_4_^*^ estimated with the sVOE/HF procedure are closer to those calculated with the SMCPP/SEP method, as compared to the sVOE/DFT counterparts. We routinely employ these two procedures, and we find the disagreement between sVOE/HF and sVOE/DFT results larger than expected. The root-mean-square deviation (RMSD) between the MP2/6–31G­(d)- and B3LYP/6–31G­(d)-optimized geometries is 0.117 Å overall, but the deviation is not uniformly distributed: the C, N, and H atoms deviate by only 0.03–0.07 Å, while the NO_2_ oxygen atoms deviate by 0.25–0.27 Å. This indicates that the structural difference is concentrated on the NO_2_ torsion angle, which, as discussed above, has a dramatic effect on the π_3_^*^ state. Furthermore, the two scaling procedures differ in their training sets and levels of theory: the sVOE/HF relation was calibrated on aromatic hydrocarbons at the HF/6–31G­(d)//MP2/6–31G­(d) level,^48^ while the sVOE/DFT relation was calibrated on phenyl-ethynyl compounds at the B3LYP/6–31G­(d) level.^49^ NDP, with its nonplanar NO_2_ group, lies outside the scope of both training sets. The observed discrepancy therefore reflects both the geometry sensitivity of the NO_2_-containing system and the inherent limitations of empirical scaling relations calibrated on simpler planar molecules. Although discrepancies are found for the π_4_^*^ state, in this case, they can arise from the mixed shape and core excited character, and also how this mixing is coupled to geometry.

Further insight into the resonance characters can be gained from the pseudo-orbitals in Figure 4. They were obtained from the pseudo-states in the SE and SEP approximations (projecting onto the SE subspace, see Computational Procedures). The σ* pseudo-orbitals exhibit antibonding character on the C–Cl bonds, indicating that electron attachment to these states should result in dissociative anion states. More importantly, there is a significant difference between the π_3_^*^ and π_4_^*^ pseudo-orbitals obtained for the NDP and *p*-NDP structures, in contrast with the other bound and transient anion states. This discrepancy is not surprising for the higher lying π_4_^*^ state, which should be sensitive to the mixing between shape and core-excited configurations and how these are affected by geometry changes. For the π_3_^*^ shape resonance, there is a clear distortion of the electronic density around the NO_2_ group as planarization is enforced (see Figure 1). The pronounced energy differences between the π_3_^*^ resonance in NDP and *p*-NDP geometries (Table 2) arise mainly from the distortion of the orientation of the nitro group, affecting the molecular dipole moment and the electron density around the NO_2_ moiety.

**Figure 4:**
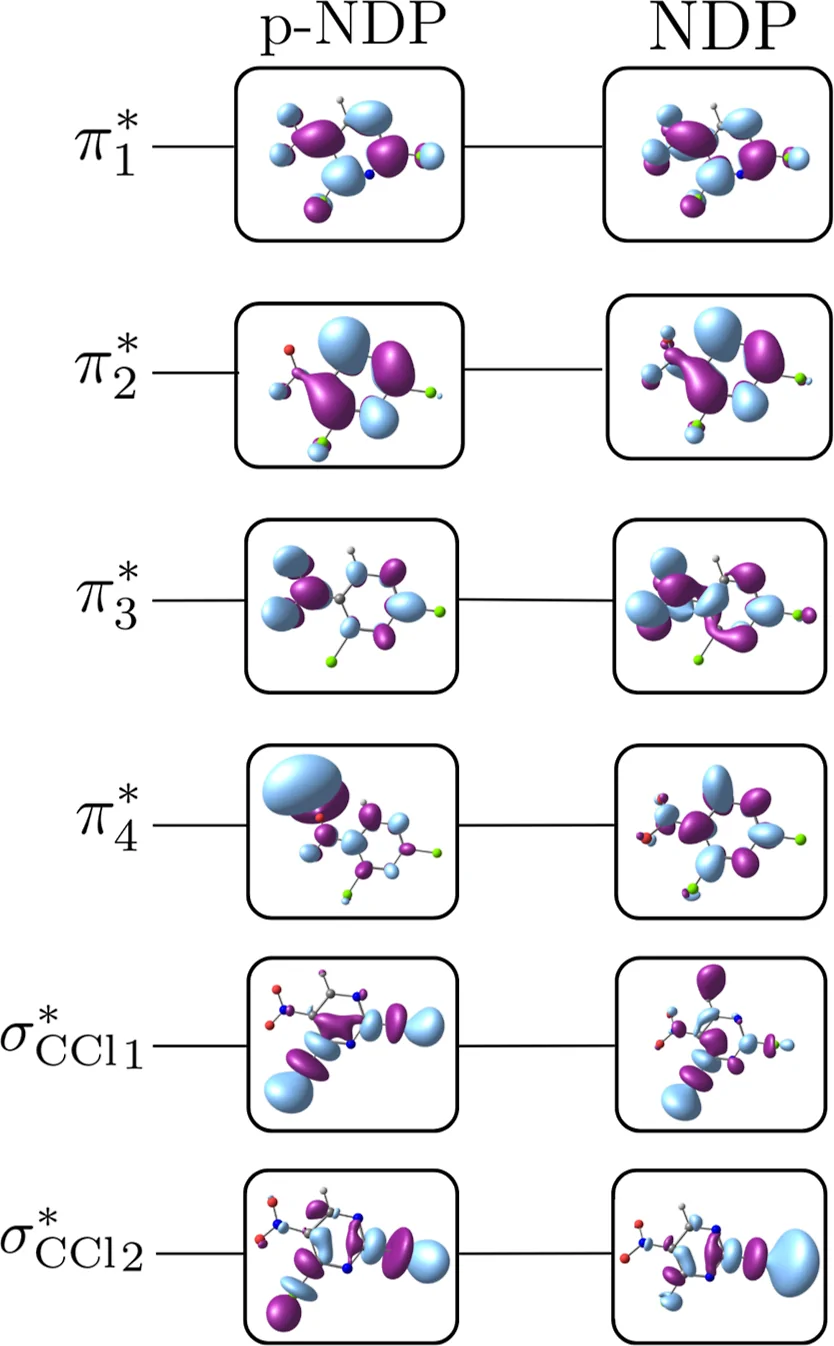
NDP and *p*-NDP pseudo-orbital isosurfaces obtained from the pseudo-eigenstates of the scattering Hamiltonian in the SEP approximation, projected onto the SE subspace. Each pseudo-orbital is a linear combination of MVOs that characterizes the excess electron in the corresponding anion state, and the isosurfaces display the sign of the wave function (phase information).

The pronounced energy shift of the π_3_^*^ resonance upon planarization, from 0.38 eV in NDP to 2.15 eV in *p*-NDP at the SMCPP/SEP level (Figure 5), is consistent with two independent pieces of evidence: (i) the anion-ground-state geometry obtained in our adiabatic electron affinity calculation is essentially planar (the Computational Procedures section), with the NO_2_ group aligned with the ring plane, and (ii) Cui et al.^54,55^ have shown, for analogous nitro-substituted pyrimidine radiosensitizers, that rotation of the NO_2_ group systematically lowers the LUMO energy and modulates DEA (“nitro-rotation-tuned DEA”). A proper description of the interplay between the NO_2_ torsion and electron correlation in π* anion states may require multiconfigurational methods.^56,57^

**Figure 5:**
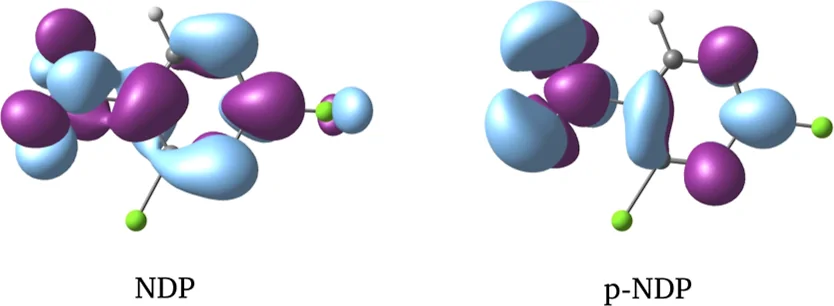
Comparison between π_3_^*^ NDP and *p*-NDP isosurfaces obtained from the ωB97XD functional with the cc-pVDZ basis set for C and H atoms and aug-cc-pVDZ for N, O, and Cl atoms.

## Discussion

The DEA process to the NDP molecule was investigated experimentally.^28^ In this experiment, the mass selected signals of the anionic fragments are observed as a function of the incident electron energy. It is worth noting that the NDP molecule was evaporated at 450 K (176.85 °C, *k*_B_ *T* ≈ 40 meV). The main fragments are reproduced in Table 3, together with the energies and relative intensities of the fragmentation signal peaks. Dissociation thresholds for zero temperature (Δ*E*) and free energy (Δ*G*) for 450 K, calculated with the G4­(MP2) method, are also shown. Remarkably, the production of all fragments is exergonic, indicating spontaneous reactions. We use the notation [M–X]^−^ to denote the anion fragment formed by releasing the neutral fragment X from the original M-molecular anion

$$M+e^{-}\to {\left[M-X\right]}^{-}+X$$

**Table 3:** Comparison of Experimental and Theoretical DEA Data for NDP^a^

fragment	energy	intensity	Δ*G* (Δ*E*)
M^–^	0.0	1.1	–1.89 (−1.84)
[M–NO]^−^	0.0	100	–3.63 (−2.91)
[M–NO_2_]^−^	0.0	75	–0.79 (0.05)
	2.0	4.9	
[NO_2_]^−^	2.8	2.3	–0.48 (0.51)
[Cl]^−^	0.15	5.4	–0.26 (0.30)
	2.7	22	–0.48 (0.09)

a Experimental peak energies and relative intensities are from Luxford et al.^28^ The dissociation free energy Δ*G* at 450 K (the temperature of the experiment) was calculated in this work using the G4­(MP2) method; the corresponding zero-temperature dissociation energy Δ*E* is given in parentheses. The two [Cl]^−^ rows correspond to the two experimentally observed Cl^–^ peaks (at 0.15 and 2.7 eV), each assigned to the dissociation of a different inequivalent C–Cl bond: the 0.15 eV peak to the C–Cl1 bond (closer to the NO_2_ group) and the 2.7 eV peak to the C–Cl2 bond (farther from it). The proposed precursor anion states and dissociation mechanisms are discussed in the Discussion section. All energies are given in eV.

The most striking feature of the experimental data is the observation of M^–^, [M–NO]^−^, and [M–NO_2_]^−^ fragments at 0 eV, with the latter two channels representing the most intense signals (Table 3). These processes, which include the formation of the parent anion (M^–^) and the production of NO and NO_2_ neutral fragments, could be initiated by vibrational Feshbach resonances (VFRs) built on weakly bound anion states, at least in principle. Considering the vibronic transition |Φ_0_ϕ_ν_0__⟩ → |Ψφ_ν_⟩, where Φ_0_ (ϕ_ν_0__) is the initial electronic (vibrational) state of the target and Ψ (φ_ν_) is the final electronic (vibrational) state of the anion formed by electron attachment, it is clear that a VFR will be formed when the kinetic energy of the projectile matches the energy difference between the |Ψφ_ν_⟩ and |Φ_0_ϕ_ν_0__⟩ states. Despite the discrepancy among the several methods used to estimate the energies of the bound π_1_^*^ and π_2_^*^ anion states (see Table 1), the higher lying π_2_^*^ state always seems too strongly bound to support the VFR mechanism. The CASPT2 method predicts the lowest binding energy for that state, −0.71 eV. Even in this case, a projectile with ∼0 eV would need to induce a transition to a high-lying vibrational state of the anion (possibly in the vibrational continuum) because the increase in vibrational energy should be ∼0.7 eV. We therefore consider VFR formation very unlikely for NDP.

The SMCPP/SEP and CASPT2 calculations describe the π_3_^*^ state as a low-lying resonance, around 0.1–0.4 eV (see Table 2). The sVOE estimates predict either a shallow bound state (−0.29 eV from sVOE/DFT) or a low-lying resonance (0.25 eV from sVOE/HF). Although a resonance energy around 0.3 eV seems too high to account for the fragmentation at ∼0 eV, we believe the following aspects should be taken into account. The balanced description of correlation-polarization effects in the SEP approximation, for a system having several resonances such as NDP, is challenging. In addition, the comparison between our SEP calculations and ETS data for several systems^23,25,27,38^ suggests that we agree with experiment within ≈0.4 eV. Finally, our calculated resonance positions are typically blue-shifted with respect to the DEA peaks. Because of these sources of inaccuracy, and also because VFR formation seems highly unlikely for NDP, we believe the π_3_^*^ state should initiate the fragmentation reactions at nearly zero energy. Here, we assume that π_3_^*^ would be either a very low-lying resonance or a very shallow bound state (our calculations would overestimate the π_3_^*^ energy).

Recalling that NDP has been suggested a universal model for radiosensitizers, we note that the unusually complex fragmentation branching at very low energies probably arises from nonadiabatic couplings between the π_3_^*^ state, expected to be formed by low-energy electron attachment, and the lower-lying bound states, π_1_^*^ and π_2_^*^. The combination of bound anion states and a low-energy resonance seems to provide a cascade for internal conversion and dissociation, which could thus be regarded as a target property to design new radiosensitizers.

Another interesting aspect is the formation of Cl^–^ fragments at a slightly higher energy, 0.15 eV. This reaction is clearly favored by the formation of σ_CCl_^*^ resonances, which would be short-lived and also lie too high in energy to initiate the process (1.42 and 2.46 eV according to the SMCPP/SEP calculations, see Table 2). One should therefore expect an indirect reaction mechanism, i.e., electron attachment to the π_3_^*^ resonance followed by nonadiabatic population transfer to the dissociative σ_CCl_^*^ states. The thermochemical threshold for this channel is exergonic at the experimental temperature (Δ*G* = −0.26 eV for the C–Cl1 bond at 450 K, see Table 3), consistent with the indirect mechanism being energetically viable. Both σ_CCl_^*^ resonances lie above π_3_^*^, by ∼1.0 eV and ∼2.1 eV for σ_CCl1_^*^ and σ_CCl2_^*^, respectively (SMCPP/SEP). Since the nonadiabatic π*/σ* coupling is favored by a smaller energy separation and, secondarily, by a larger orbital overlap,^58^ both criteria identify σ_CCl1_^*^ as the preferred acceptor: it has the smaller π_3_^*^ energy gap and the larger spatial overlap with π_3_^*^ among the NDP pseudo-orbitals (Figure 4). In any case, the DEA peak at 0.15 eV suggests electron attachment accompanied by vibrational excitation, such that C–Cl stretch vibrations are expected to stabilize the σ* resonances, thus favoring the π_3_^*^/σ_CCl_^*^ coupling. The vibrational analysis performed with the ωB97XD/cc-pVDZ method indicates three normal modes with significant C–Cl components. The fundamental vibrational energies are 0.005, 0.011, and 0.150 eV, which we find compatible with the DEA data.

Since the σ_CCl_^*^ resonances have low electronic densities around the NO_2_ group (see Figure 4) and are short-lived (see Table 2), they seem unlikely to initiate the formation of NO_2_^–^ around 2.8 eV. The Cl^–^ signal at 2.7 eV could be initiated by the σ_CCl2_^*^ resonance (computed at 2.46 eV in the SEP approximation) and/or by the mixed-character π_4_^*^ resonance, whose SEP energy is expected to downshift by ∼1 eV toward this region once electronic excitation channels are included in the calculation.^23,59,60^ In this region, the two become near-degenerate and can undergo strong resonance–resonance coupling, which is known to modify DEA cross sections and, for short-lived resonances, to operate through the nuclear motion;^58,61^ for σ*/π* resonances of a chlorinated anion, it can even produce intersecting complex potential energy surfaces.^62^ Unlike the comparatively isolated σ_CCl1_^*^, whose energy hosts no long-lived π* partner and near which no Cl^–^ band is observed (1.4 eV), σ_CCl2_^*^ therefore cannot be excluded as an initiator within our fixed-nuclei framework. The dissociation threshold for this channel (Δ*G* = −0.48 eV for the C–Cl2 bond at 450 K, Table 3) is also exergonic. On the other hand, the production of the [M–NO_2_]^−^ fragment around 2.0 eV has no simple interpretation based on our fixed-nuclei calculations. We also note that higher-lying resonances could in principle decay via neutral dissociation through a catalytic electron mechanism, as recently reported for a related nitroimidazole radiosensitizer.^63^

The qualitative picture underlying both the direct and the indirect DEA mechanisms discussed above is further illustrated in the Supporting Information by one-dimensional rigid PES scans along the C–Cl and C–N­(NO_2_) bonds, which show that an anion state acquiring appreciable σ*­(C–X) antibonding character is steeply stabilized upon bond elongation and therefore drives fragmentation. These scans are meant as a qualitative visualization only; a quantitative description of the dissociation dynamics would require nonadiabatic surface hopping on multireference PESs.^64,65^

## Conclusions

We reported a theoretical investigation of the low-energy anion states of the compound NDP. The molecule presents a unique combination of the mechanisms usually credited with the action of radiosensitizers, pointing to a new paradigm in which drugs may have several mechanisms underlying their biological activity. Our calculations show that NDP supports two distinct dissociation routes leading to Cl^–^ formation. In the direct route, the incoming electron is captured by a dissociative σ_CCl_^*^ resonance, whereas in the indirect route, it is initially trapped in a π* resonance and subsequently transferred to the σ_CCl_^*^ channel through π*/σ* coupling. This interplay between direct σ* capture and π*-mediated, intramolecular electron transfer to the C–Cl bond is well documented for halogenated aromatics^66,67^ and underlies the variety of fragment channels expected for NDP under low-energy electron impact, relevant to its proposed role as a radiosensitizer model.^68^ Our protocol, spanning bound-state calculations employing both TDDFT and MS-CASPT2 methods, electron-molecule scattering techniques, and dissociation threshold analysis, identifies five key electronic states: two bound anions (π_1_^*^ and π_2_^*^), three shape resonances (π_3_^*^, σ_CCl1_^*^, and σ_CCl2_^*^), and a π_4_^*^ resonance of mixed shape and core-excited character.

We routinely resort to planarization to help the interpretation of the transient anion spectra of small biomolecules. However, we find the energy discrepancies between the NDP and *p*-NDP structures unusually large. Our results show that small geometric modifications could alter both direct and indirect DEA mechanisms. In particular, the pronounced energy shift of the π_3_^*^ resonance upon planarization (from 0.38 eV in NDP to 2.15 eV in *p*-NDP) demonstrates that simplified planar models fail to capture the essential geometric sensitivity of NO_2_-containing systems. We believe this behavior stems from the electronic density around the NO_2_ site.

We attribute the complex fragmentation—[M–NO]^−^ at 0 eV—to the π_3_^*^ state. Our calculations, which employed several methods, characterize that state either as a weakly bound state or a low-lying resonance (−0.29 to 0.38 eV). While such processes could in principle occur via VFRs, given the presence of two bound anion states, our findings suggest that the π_2_^*^ state is too deeply bound to enable this mechanism. Even the most favorable estimate of the binding energy (−0.71 eV) would still require implausibly high vibrational excitation, making the VFR formation in NDP unlikely. Instead, the intricate fragmentation patterns should stem from nonadiabatic interactions between the π_3_^*^ state, formed during low-energy electron capture, and the lower-lying bound states, π_1_^*^ and π_2_^*^. This interplay between bound anion states and a low-energy resonance creates pathways for internal conversion and dissociation, a feature that could be exploited to design novel radiosensitizers.

Additionally, the emergence of Cl^–^ fragments at slightly higher energies (0.15 eV) points to an indirect mechanism, where electron attachment to the π_3_^*^ resonance is followed by nonadiabatic transitions to dissociative σ_CCl_^*^ states. The pseudo-orbital analysis identifies σ_CCl1_^*^ as the primary acceptor, owing primarily to its smaller energy separation from π_3_^*^, supported by the larger spatial overlap of the corresponding pseudo-orbitals, in the NDP geometry. Lastly, the mixed-character π_4_^*^ and the σ_CCl2_^*^ shape resonances are expected to drive fragmentation near 2.7 eV. In contrast, the appearance of the [M–NO_2_]^−^ fragment around 2.0 eV lacks a straightforward explanation within our fixed-nuclei framework. Nuclear dynamics simulations on the anion potential energy surfaces, which go beyond the fixed-nuclei approximation employed here, would be required to confirm the proposed nonadiabatic dissociation pathways and to elucidate the origin of this fragment.

## Supplementary Material



## Data Availability

are available in the Supporting Information. The SMCPP code is codeveloped by several groups in Brazil and elsewhere; the policy of this group of codevelopers is sharing the code by request, not keeping public versions, and it can be obtained upon reasonable request to the corresponding authors. The input-preparation and analysis scripts used in this work are publicly available at https://github.com/elyymiranda/SMC2023.

## Notes

Optimized Cartesian coordinates of all stationary points (NDP and *p*-NDP geometries), complete active space molecular orbitals, anion binding energies calculated with different DFT functionals, empirical scaling relations employed to estimate vertical attachment energies from virtual orbital energies, the Cartesian–Gaussian basis sets used in the scattering calculations, and potential energy surface scans along the C–Cl and C–NO_2_ dissociation coordinates (PDF)
