# Supplementary material for: Anion States and Dissociation Pathways of the Universal Radiosensitizer Model 2,4-Dichloro-5-nitropyrimidine
Source: J Phys Chem A. 2026 Aug 10;130(33):6518–27. doi: 10.1021/acs.jpca.6c00754 (PMC13501881; doi:10.1021/acs.jpca.6c00754)
Supplement: Supplementary file 1 [file jp6c00754_si_001.pdf]

Supporting Information: Anion states and dissociation pathways of the universal radiosensitizer model 2,4-dichloro-5-nitropyrimidine

Ely G. F. de Miranda* and Márcio T. do N. Varella*

Instituto de Física, Universidade de São Paulo, Rua do Matão 1371, 05508-090, São Paulo, São Paulo, Brazil

E-mail: ely.miranda@usp.br; mvarella@if.usp.br

Anion binding energies

The binding energies were calculated employing DFT/aug-cc-pVDZ methodology with several functionals, as shown in Tab. S1, with the geometry optimized with the ωB97XD functional with the same basis. All the functionals confirm the anion-bound state, with large binding energies of ≈ 1.6 eV and ≈ 1.1 eV for NDP and p-NDP, respectively. A dipole-bound state (DBS) existence was also investigated; however, we could not infer the formation of any DBS as already expected, since the dipole moment magnitude is considered small for this type of state.[S1]

Table S1: Binding energy (BE, in eV) and Dipole Moment (μ, in Debye) estimations for p-NDP and NDP.

Functional	BE(p-NDP)	BE(NDP)	μ(p-NDP)	μ(NDP)
ωB97XD	-1.05	-1.57	0.78	1.10
B3LYP	-1.15	-1.69	0.84	1.19
CAM-B3LYP	-1.13	-1.67	0.83	1.15
M06-2X	-1.04	-1.57	0.87	1.21

CASSCF/CASPT2 neutral and anion states

The vertical energies of the anion states of NDP and p-NDP were computed with the SA-CASSCF and MS-CASPT2 method and the cc-pVDZ basis set for H and C atoms and the aug-cc-pVDZ basis for N, O, and Cl atoms employing the OpenMolcas software.[S2] The CASSCF wave function was state-averaged over seven roots and point-group symmetry was not enforced during the orbital optimization. The neutral ground state of each molecule was computed at the same level of theory and within the same state-averaging framework, and serves as the zero-point reference for the vertical anion-state energies; the neutral excited states obtained as a byproduct of this protocol are not interpreted as observables in this work. The CASSCF active space was constructed following the bonding–antibonding pairing principle. The six valence anion states identified in this work, namely three π^* (π_1^*,

π^*_2, π^*_3), two σ^*_{CCl} (σ^*_{CCl1}, σ^*_{CCl2}), and one higher-lying π^*_4, define six virtual orbitals that must be included. Each is paired with its occupied correlating orbital of matching character (the corresponding bonding π or σ_{CCl} orbital), yielding 12 orbitals. Additionally, the $\sigma^*_{\mathrm{C\text{–}NO_2}}$ antibonding orbital and its bonding counterpart $\sigma_{\mathrm{C\text{–}NO_2}}$ were included, as this pair describes the C–NO_2 dissociation coordinate relevant to the prominent $[\mathrm{M\text{–}NO}]^-$ and $[\mathrm{M\text{–}NO_2}]^-$ fragmentation channels. The resulting initial guess of the active space comprises 7 doubly occupied and 7 unoccupied orbitals, yielding a (14e,14o) active space for the *neutral* molecule. For the *anion*, the same 14 active orbitals are retained and one electron is added, leading to a (15e,14o) active space. The neutral and the anion were treated as two independent SA-CASSCF calculations, each variationally optimizing its own active orbitals for the corresponding charge state; the converged neutral orbitals were used only as the initial guess for the anion, whose orbitals were then fully re-optimized, so that no orbitals were frozen or transferred unrelaxed between the two calculations. In the anion, the additional electron is accommodated within the active space, and the SA-CASSCF ground state is dominated by a configuration in which a natural orbital of ring π^* character (labeled π^*_1) carries this electron, its state-averaged natural occupation number being close to one. The molecular orbitals and their characters are indicated in Figs. S1 and S2 for NDP and p-NDP, respectively. In the CASPT2 step, no IPEA shift was used (IPEA = 0)[S3] and an imaginary level shift of 0.20 a.u. was applied to remove intruder states.[S4] In the planar p-NDP geometry the σ and π orbitals separate by symmetry and the C–NO_2 σ/σ^* pair is well localized (Fig. S2); in NDP the out-of-plane rotation of the nitro group removes this local symmetry, so the C–NO_2 σ character delocalizes over the neighboring nitro (N–O) and C–Cl in-plane orbitals.

Empirical scaling relations for virtual orbital energies

The energies of the bound anion states and shape resonances reported in Table 2 of the main text were also estimated from empirically corrected virtual orbital energies (VOEs) through

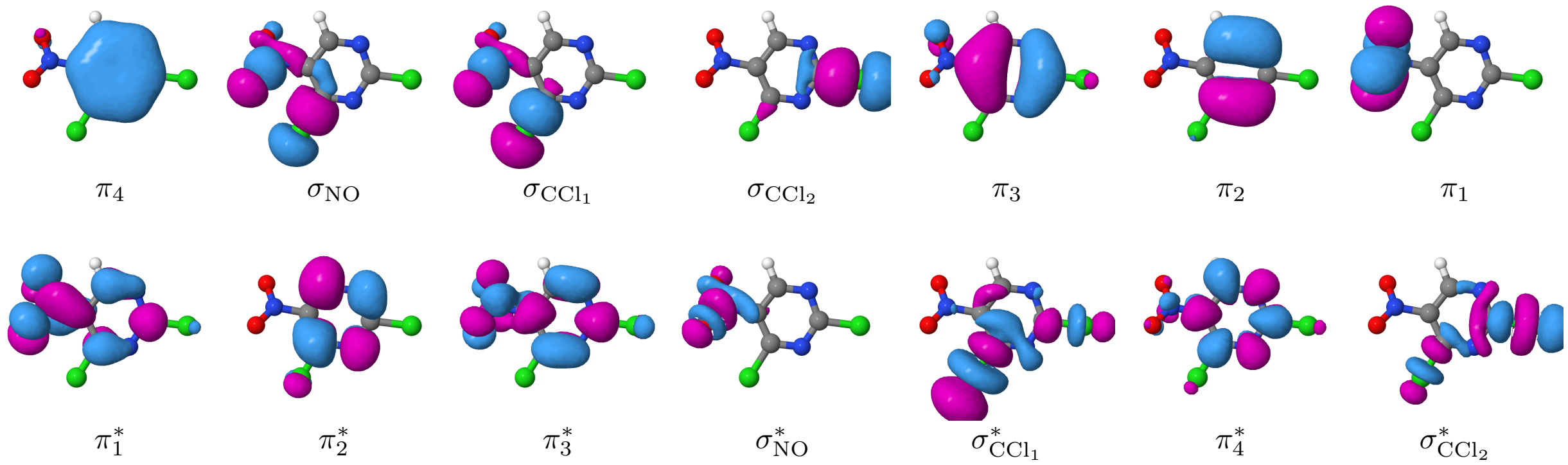

Figure S1: Active-space orbitals of the SA-CASSCF calculation for NDP, shown as the converged state-averaged natural orbitals and labeled by their dominant character. The wave function is state-averaged over seven roots and point-group symmetry was not enforced during the orbital optimization.

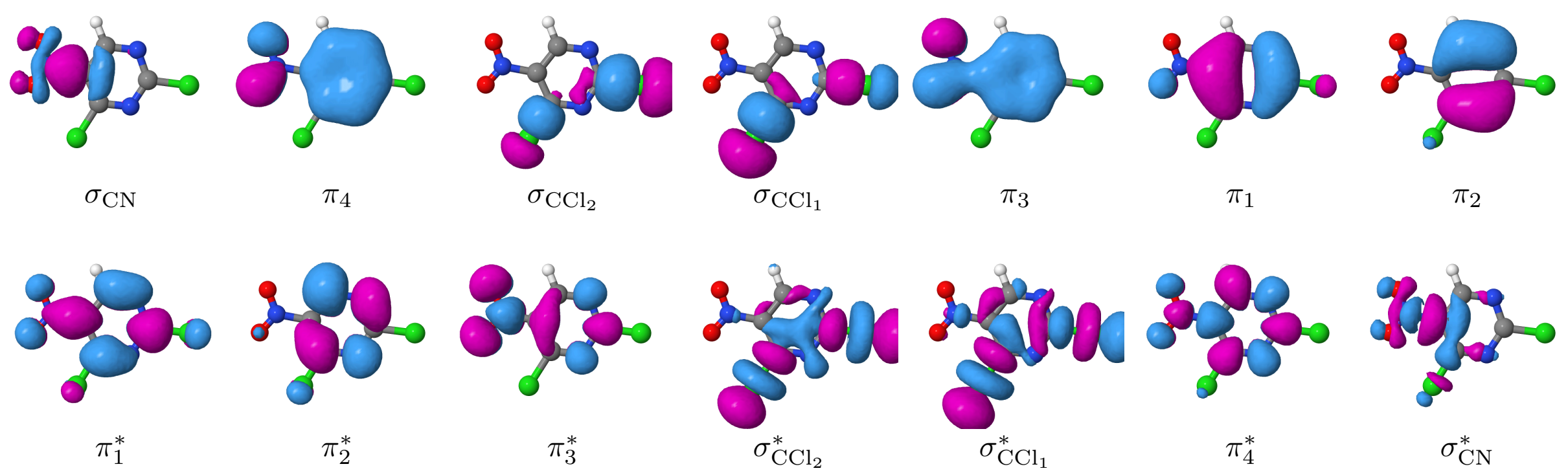

Figure S2: Active-space orbitals of the SA-CASSCF calculation for p-NDP, shown as the converged state-averaged natural orbitals and labeled by their dominant character. The wave function is state-averaged over seven roots and point-group symmetry (C_s) was deliberately not enforced during the orbital optimization, so that NDP and p-NDP could be treated within the same framework; the planar geometry nonetheless yields a clean σ/π separation, and the C–NO_2 σ/σ^* pair is well localized.

the well-established scaled-VOE (sVOE) methodology. This approach relies on a linear correlation of the form VAE $= A \times$ VOE $+ B$ between the calculated VOEs of the neutral molecule (in eV) and the vertical attachment energies (VAEs) measured experimentally via electron transmission spectroscopy for a training set of related compounds. The coefficients A and B are specific to the level of theory and the geometry optimization protocol used to generate the VOEs; consequently, each scaling relation must be applied strictly within its

original combination of method and geometry.

In this work we employed four distinct scaling relations, organized by orbital character (π^* or σ^*_{CCl}) and level of theory (HF or DFT). The equations and references are summarized in Table S2, where the "HF" label refers to the HF/6-31G(d)//MP2/6-31G(d) protocol (VOEs computed at HF/6-31G(d) on MP2/6-31G(d) optimized geometries) and the "DFT" label refers to the B3LYP/6-31G(d) protocol (both geometry optimization and VOEs at B3LYP/6-31G(d)).

Table S2: Empirical scaling relations employed in this work.

Orbital	Method	Scaling relation (eV)	Reference
π^*	HF	VAE = 0.648 VOE − 1.430	Modelli (2004)[S5]
π^*	DFT	VAE = 0.807 VOE + 0.919	Scheer and Burrow (2006)[S6]
σ^*_{CCl}	HF	VAE = 0.900 VOE − 2.550	Aflatooni *et al.* (2000)[S7]
σ^*_{CCl}	DFT	VAE = 0.811 VOE + 1.610	Modelli *et al.* (2008)[S8]

The π^* scaling relations of Modelli (2004)[S5] and Scheer and Burrow (2006)[S6] were calibrated on training sets of aromatic hydrocarbons and phenyl–ethynyl compounds, respectively, and are standard choices in the ETS literature for π^* resonances of unsaturated molecules. The σ^*_{CCl} scaling relations of Aflatooni *et al.* (2000)[S7] and Modelli *et al.* (2008)[S8] were specifically derived from mono- and poly-chloroalkane ETS data and are therefore the appropriate choice for the σ^*_{CCl} orbitals of NDP. Because each scaling combines a specific level of theory with a specific geometry optimization protocol, both MP2/6-31G(d) and B3LYP/6-31G(d) optimizations of NDP and p-NDP were performed. The corresponding Cartesian coordinates are listed in Tables S3–S6 below. All other calculations reported in the manuscript were carried out at the ωB97XD/aug-cc-pVDZ geometry.

Scattering details

The scattering calculations were carried out using the SMCPP method implemented in a group-developed algorithm that has been constructed and refined over the last decades.

Table S3: NDP geometry optimized at MP2/6-31G(d) (in angstrom).

Atom	X	Y	Z
C	−0.042114340746	−1.630199890682	0.051619818000
C	0.256956289925	0.738312722814	−0.039447836697
C	0.819202413131	−0.538790007733	0.024098284183
C	−1.803577947596	−0.219700094886	0.018257664449
Cl	1.202650650801	2.162736568529	−0.170648728942
Cl	−3.509639397502	0.007473965001	0.040464924630
H	0.353768462222	−2.642720817824	0.070377139757
N	−1.066495170295	0.891763430352	−0.046033153815
N	−1.372268675815	−1.484539312554	0.059018474993
N	2.253215511526	−0.797608499385	0.059493948159
O	2.661238978770	−1.717030638098	−0.669953124237
O	2.936396024364	−0.109957357975	0.833481255758

Table S4: NDP geometry optimized at B3LYP/6-31G(d) (in angstrom).

Atom	X	Y	Z
C	−0.034768247353	−1.624483770956	0.023949489224
C	0.251906183159	0.743781028834	−0.027500173207
C	0.836061338812	−0.533011714972	0.013177798660
C	−1.794471696158	−0.230446123211	0.014515944463
Cl	1.163593935464	2.209101380428	−0.142788700389
Cl	−3.516640184960	−0.010716519232	0.042501465707
H	0.369213398777	−2.632312525765	0.031139690281
N	−1.066609948840	0.880404307320	−0.029673111466
N	−1.359445922597	−1.488391294293	0.033367732561
N	2.275677201875	−0.810476031582	0.049896425074
O	2.633227076346	−1.881213044370	−0.440107758493
O	3.008380185465	0.012959854682	0.584326212728

The SMCPP code is co-developed by several groups in Brazil and elsewhere; the policy of this group of co-developers is sharing the code by request, not keeping public versions, and it can be obtained upon reasonable request to the corresponding authors. The input-preparation and analysis scripts used in this work are publicly available at `https://github.com/elyymiranda/SMC2023`. The NDP and p-NDP geometries employed in the scattering calculation were presented in Tab. S7 and S8 respectively.

The exponents employed in the atom basis for the scattering calculation are shown in Tabs. S9 and S10.

Table S5: **p-NDP geometry optimized at MP2/6-31G(d) (in angstrom).**

Atom	X	Y	Z
C	−0.507868813680	−1.541818638051	0.000000000000
C	0.000000000000	0.793561087196	0.000000000000
C	−0.966028504150	−0.223489397168	0.000000000000
C	1.622146998309	−0.810850144021	0.000000000000
Cl	−0.332361641209	2.475294073464	0.000000000000
Cl	3.305005110660	−1.170092703050	0.000000000000
H	−1.219504692177	−2.361140909829	0.000000000000
N	1.298769030449	0.480533138925	0.000000000000
N	0.790765888299	−1.857900685718	0.000000000000
N	−2.425560487448	−0.023903382645	0.000000000000
O	−3.121717050860	−1.050889415715	0.000000000000
O	−2.859877115869	1.135538995055	0.000000000000

Table S6: p-NDP geometry optimized at B3LYP/6-31G(d) (in angstrom).

Atom	X	Y	Z
C	−0.495191020632	−1.545396035007	0.000000000000
C	0.000000000000	0.789962205644	0.000000000000
C	−0.970901882648	−0.229543371696	0.000000000000
C	1.620283325998	−0.800715304306	0.000000000000
Cl	−0.347222600507	2.483609178455	0.000000000000
Cl	3.320291570271	−1.152433608450	0.000000000000
H	−1.205421877969	−2.365349165442	0.000000000000
N	1.291945718456	0.485075606485	0.000000000000
N	0.800424844206	−1.850009097154	0.000000000000
N	−2.430769443698	−0.040130812011	0.000000000000
O	−3.112431106704	−1.065265769835	0.000000000000
O	−2.874206510799	1.100886971219	0.000000000000

Potential energy surface scans along dissociation coordinates

To provide a qualitative visualization of the dissociative character of the anion states discussed in the main text, we performed rigid scans of the potential energy surfaces (PESs) of NDP along the three bonds most relevant to the experimentally observed DEA channels: the two C–Cl bonds (C2–Cl1 and C4–Cl2) and the C–N bond that links the pyrimidine ring to the nitro group (C3–N of NO_2). For each coordinate, 19 geometries were generated by

Table S7: NDP geometry (in angstrom).

Atom	X	Y	Z
C	0.037930058873	1.630331041971	0.029067533776
C	-0.251284603096	-0.741168020372	-0.029274242051
C	-0.831845595341	0.537286223578	0.017850671052
C	1.797283998065	0.228903043941	0.016007956806
Cl	-1.175511562748	-2.200118347913	-0.157914208093
Cl	3.521416030799	0.006384757935	0.044353290110
H	-0.365597651816	2.643008700831	0.040340079705
N	1.067199886221	-0.881627410571	-0.033771317845
N	1.363368237285	1.487750905153	0.038852325916
N	-2.274556478547	0.806593727986	0.056416190836
O	-2.653131485888	1.822413710143	-0.522194181571
O	-2.986788880564	0.031252291262	0.679419634254

Table S8: p-NDP geometry (in angstrom).

Atom	X	Y	Z
C	0.037930058873	1.630331041971	0.000000000000
C	-0.251284603096	-0.741168019843	0.000000000000
C	-0.831845594811	0.537286224107	0.000000000000
C	1.797283998065	0.228903043941	0.000000000000
Cl	-1.175511563277	-2.200118347913	0.000000000000
Cl	3.521416030799	0.006384757935	0.000000000000
H	-0.365597651816	2.643008700831	0.000000000000
N	1.067199886221	-0.881627411100	0.000000000000
N	1.363368236756	1.487750905153	0.000000000000
N	-2.274556479076	0.806593727986	0.000000000000
O	-2.653131485888	1.822413710143	0.000000000000
O	-2.986788881093	0.031252291262	0.000000000000

displacing the selected bond in steps of 0.05 Å around the ωB97XD/aug-cc-pVDZ equilibrium structure used throughout the manuscript, with all remaining internal coordinates kept frozen. At every geometry, single-point calculations of the closed-shell neutral (charge 0, singlet) and of the open-shell anion (charge −1, doublet) were carried out at the ωB97XD level using cc-pVDZ for C and H atoms and aug-cc-pVDZ for N, O, and Cl atoms. Five excited states of the anion (D_1–D_5) were computed on top of the anion ground state (D_0) within the linear-response TDDFT framework, and the neutral ground state (S_0) at the equilibrium geometry was taken as the zero of the energy axis.

Table S9: Cartesian-Gaussian basis sets employed in the scattering calculations for Carbon, Oxygen, Nitrogen, and Chlorine atoms. The exponents are given in atomic units.

Type	Carbon	Oxygen	Nitrogen	Chlorine
s	12.49408	16.05878	17.56734	10.49065
s	2.47029	5.92024	3.423615	6.836599
s	0.61402	1.03490	0.884301	2.420592
s	0.18402	0.31684	0.259045	0.513579
s	0.03998	0.06520	0.055708	0.188863
s				0.062954
p	5.22886	10.14120	7.050692	6.037205
p	1.59205	2.78299	1.910543	2.012401
p	0.56861	0.84100	0.579261	0.686842
p	0.21032	0.23293	0.165395	0.218056
p	0.07225	0.07764	0.037192	0.071193
d	0.60359	0.75679	0.403039	1.611766
d	0.15675	0.18075	0.091192	0.328314

Table S10: Cartesian-Gaussian basis set employed in the scattering calculations for the Hydrogen atom. The exponents are given in atomic units.

Type	Exponent	Coefficient
s	13.3615	0.130844
s	2.0133	0.921539
s	0.4538	1.000000
s	0.1233	1.000000

The three panels of Fig. S3 show the resulting adiabatic curves for the atoms labeled in Fig. S4. At the neutral equilibrium geometry, D_0–D_2 have predominantly ring-localized π^* character (in line with the bound π_1^*, π_2^*, and the low-lying π_3^* shape resonance of the main text), whereas D_3 has predominantly antibonding σ^*(C–X) character along the scanned bond and lies higher in energy, as expected for an antibonding orbital at short bond distance. Taking panel (a) as a guiding example: as the C–Cl1 bond is elongated, the σ^*_{CCl1} character is steeply stabilized (a natural consequence of relaxing antibonding nodal structure upon bond lengthening) and, in the adiabatic picture, this stabilization manifests itself as a sequence of avoided crossings. Near the first avoided crossing, D_3 and D_2 exchange orbital character, so that D_2 acquires σ^* character while the π^* character previously carried by D_2 is pushed

up into D_3; further elongation triggers additional avoided crossings with the remaining π^* states. The antibonding character is thus progressively transferred to the lower adiabatic states as the bond is stretched, and panels (b) and (c) display the same qualitative behaviour along the C4–Cl2 and C3–N(NO_2) coordinates.

The central physical content of the figure is the following: once an anion state acquires appreciable σ^*(C–X) antibonding character at a given geometry, the corresponding diabatic curve is steeply stabilized upon bond elongation, so that the associated nuclear motion naturally drives the system towards fragmentation. Crucially, this conclusion is independent of how the σ^* character is populated: a shape resonance with dominant σ^* character at the neutral equilibrium geometry can be populated directly by vertical electron attachment and dissociate on the same diabatic curve (*direct DEA*); alternatively, a ring-localized π^* state can be populated first and then transfer its population to a σ^*-like state through non-adiabatic coupling in the avoided-crossing regions, with dissociation proceeding on the accepting state (*indirect DEA*). In both cases the fragmentation can take place, and a quantitative description would require nonadiabatic nuclear dynamics on coupled anion PESs, well beyond the scope of the present fixed-nuclei study.

We stress, accordingly, that the scans reported here are intended as a qualitative visualization of the dissociative topology of the anion states and not as a quantitative prediction of dissociation energetics or branching ratios. Single-reference TDDFT is known to have limited accuracy for anion states, particularly far from the equilibrium geometry, where the multiconfigurational character of the wave function grows, and a fully quantitative description of the dissociation dynamics would require nonadiabatic surface hopping simulations on multireference PESs.[S9] TDDFT has nonetheless been shown to remain sufficient for the qualitative description of excited-state topologies relevant to photophysical relaxation,[S9] and the feature that matters for the mechanistic discussion of the main text, namely the steep stabilization of σ^*(C–X) antibonding character upon bond elongation and the consequent downhill character of the associated excited-state PES, is already robustly captured at this

level.

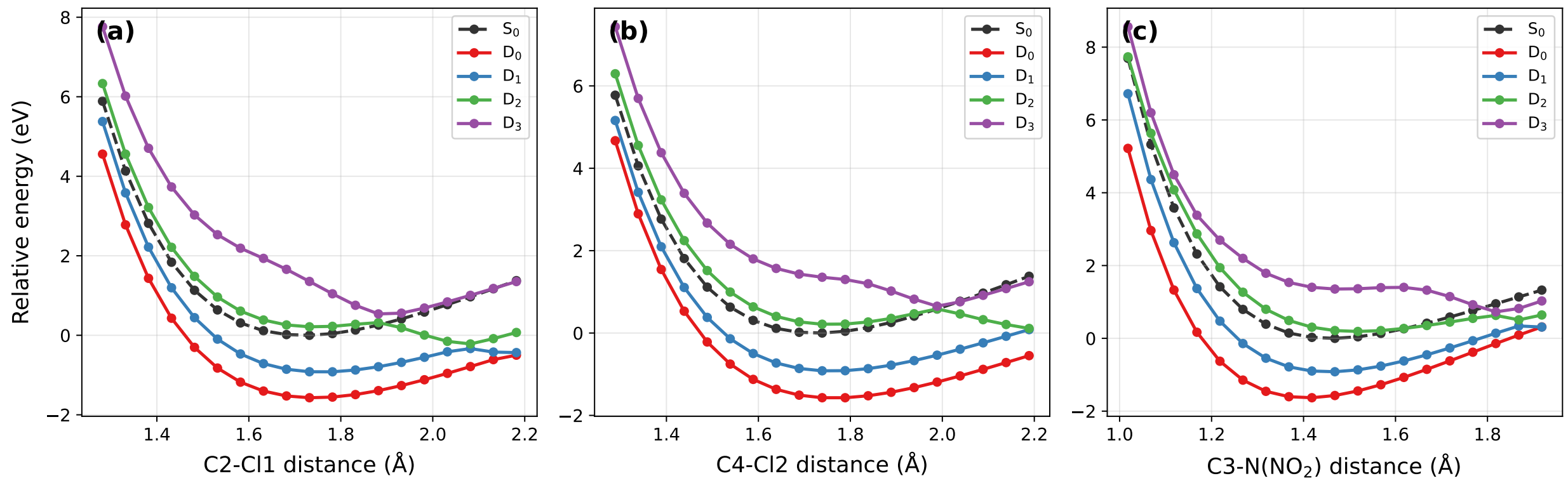

Figure S3: Adiabatic potential energy curves along the three dissociation coordinates of NDP relevant to the observed DEA channels: (a) C2–Cl1 stretching, (b) C4–Cl2 stretching, and (c) C3–N(NO_2) stretching. Each panel shows the neutral ground state S_0 (grey dashed), the anion ground state D_0 (red), and the three lowest anion excited states D_1 (blue), D_2 (green), and D_3 (purple). The energy zero corresponds to S_0 at the equilibrium geometry. Calculations were performed at the ωB97XD level using cc-pVDZ for C and H atoms and aug-cc-pVDZ for N, O, and Cl atoms, on 19 rigid geometries generated by displacing the scanned bond in steps of 0.05 Å around the ωB97XD/aug-cc-pVDZ equilibrium structure. The curves shown are adiabatic and therefore do not cross; wherever two states of distinct orbital character would cross in a diabatic representation, they instead develop an avoided crossing along which the orbital character is exchanged continuously. At the equilibrium geometry, D_0–D_2 have predominantly π^* character (ring-localized, quasi-bound along the scanned bonds), whereas D_3 has predominantly σ^*(C–X) antibonding character along the scanned bond. As the bond is elongated, the antibonding character is steeply stabilized and migrates from D_3 into the lower adiabatic states through a sequence of avoided crossings, so that dissociation naturally takes place on whichever excited adiabatic state carries the σ^* character at the relevant bond distance, without the need to first reach the anion ground state.

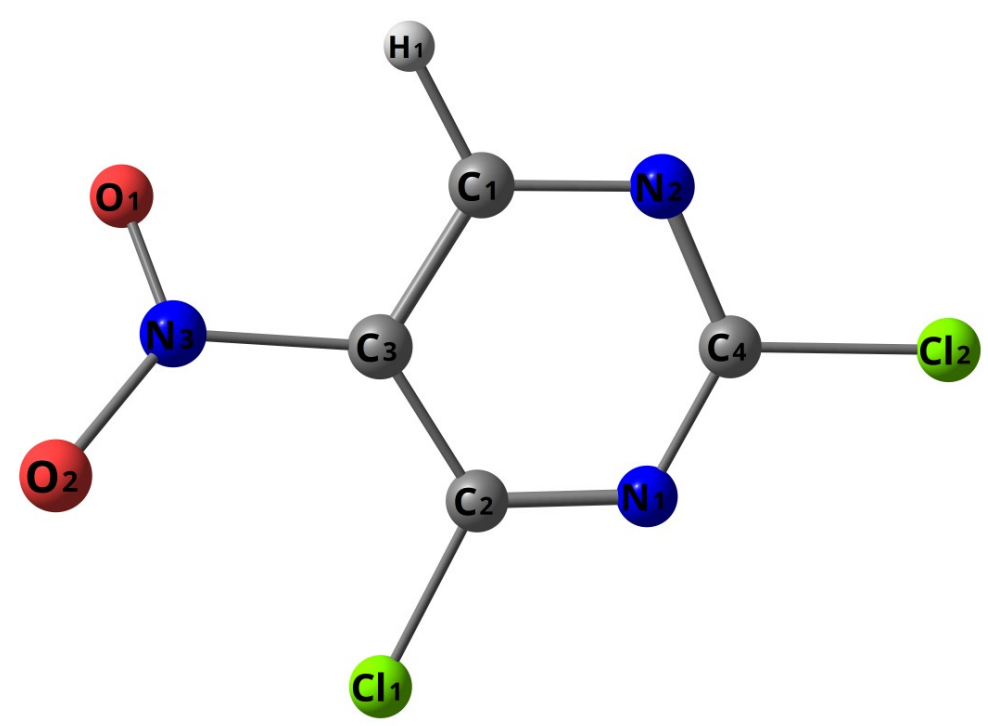

Figure S4: Numbered geometry of NDP employed as the first step in PES calculation.

References

(S1) Desfrançois, C.; Abdoul-Carime, H.; Schermann, J.-P. Ground-state dipole-bound anions. *International Journal of Modern Physics B* **1996**, *10*, 1339–1395.

(S2) Fdez. Galván, I.; Vacher, M.; Alavi, A.; Angeli, C.; Aquilante, F.; Autschbach, J.; Bao, J. J.; Bokarev, S. I.; Bogdanov, N. A.; Carlson, R. K.; Chibotaru, L. F.; Creutzberg, J.; Dattani, N.; Delcey, M. G.; Dong, S. S.; Dreuw, A.; Freitag, L.; Frutos, L. M.; Gagliardi, L.; Gendron, F.; Giussani, A.; González, L.; Grell, G.; Guo, M.; Hoyer, C. E.; Johansson, M.; Keller, S.; Knecht, S.; Kovacevic, G.; Källman, E.; Li Manni, G.; Lundberg, M.; Ma, Y.; Mai, S.; Malhado, J. P.; Malmqvist, P. Å.; Marquetand, P.; Mewes, S. A.; Norell, J.; Olivucci, M.; Oppel, M.; Phung, Q. M.; Pierloot, K.; Plasser, F.; Reiher, M.; Sand, A. M.; Schapiro, I.; Sharma, P.; Stein, C. J.; Sørensen, L. K.; Truhlar, D. G.; Ugandi, M.; Ungur, L.; Valentini, A.; Vancoillie, S.; Veryazov, V.; Weser, O.; Wesolowski, T. A.; Widmark, P.-O.; Wouters, S.; Zech, A.; Zobel, J. P.; Lindh, R. OpenMolcas: From source code to insight. *Journal of Chemical Theory and Computation* **2019**, *15*, 5925–5964.

(S3) Zobel, J. P.; Nogueira, J. J.; González, L. The IPEA dilemma in CASPT2. *Chemical Science* **2017**, *8*, 1482–1499.

(S4) Forsberg, N.; Malmqvist, P.-Å. Multiconfiguration perturbation theory with imaginary level shift. *Chemical Physics Letters* **1997**, *274*, 196–204.

(S5) Modelli, A.; Burrow, P. D. Electron Attachment to the Aza-Derivatives of Furan, Pyrrole, and Thiophene. *The Journal of Physical Chemistry A* **2004**, *108*, 5721–5726.

(S6) Scheer, A. M.; Burrow, P. D. π^* Orbital system of alternating phenyl and ethynyl groups: Measurements and calculations. *The Journal of Physical Chemistry B* **2006**, *110*, 17751–17756.

(S7) Aflatooni, K.; Gallup, G. A.; Burrow, P. D. Temporary Anion States of Dichloroalkanes and Selected Polychloroalkanes. *The Journal of Physical Chemistry A* **2000**, *104*, 7359–7369.

(S8) Modelli, A.; Burrow, P. D.; Gallup, G. A. Are There π^* Shape Resonances in Electron Scattering from Phosphate Groups? *The Journal of Physical Chemistry A* **2008**, *112*, 4106–4113.

(S9) Miranda, E. G. F. d.; Mattos, R. S.; Mukherjee, S.; Toldo, J. M.; Choi, C. H.; Varella, M. T. d. N.; Barbatti, M. Surface Hopping with Fully Correlated Methods. *Journal of Chemical Theory and Computation* **2026**, *22*, 1–19.
